# Relationship between the Length of Sperm Tail Mitochondrial Sheath and Fertility Traits in Boars Used for Artificial Insemination

**DOI:** 10.3390/antiox9111033

**Published:** 2020-10-23

**Authors:** Karl Kerns, Jennifer Jankovitz, Julie Robinson, Amanda Minton, Chris Kuster, Peter Sutovsky

**Affiliations:** 1 Division of Animal Sciences, University of Missouri, Columbia, MO 65211-5300, USA; kkh55@umsystem.edu (K.K.); jljankovitz@ucdavis.edu (J.J.); 2 Department of Animal Science, Iowa State University, Ames, IA 50011, USA; 3 Department of Animal Science, University of California-Davis, Davis, CA 65316, USA; 4 Kuster Research and Consulting, Inc., Atkinson, IL 61235, USA; julie@kusterresearch.com (J.R.); ckuster@kusterresearch.com (C.K.); 5 Acuity Genetics, Carlyle, IL 62231, USA; amanda.minton@acuityswine.com; 6 The Maschhoffs, LLC, Carlyle, IL 62231, USA; 7 Department of Obstetrics, Gynecology and Women’s Health, University of Missouri, Columbia, MO 65211-5300, USA

**Keywords:** fertility, biomarker, aggresome, ubiquitin, mitochondria

## Abstract

The length of sperm tail midpiece, occupied by the mitochondrial sheath (MS), has been correlated with reproductive traits of mice, fish, and birds; however, it is not known whether such a correlation exists in higher order species such as domestic pigs. As the mitochondria provide for sperm motility and generate the fertility-affecting reactive oxygen species (ROS), we hypothesized that MS length correlates with boar semen parameters and artificial insemination (AI) fertility. Sperm samples collected from 57 boars and used for single sire AI were labeled with ProteoStat Aggresome probe (AGG; Enzo Life Sciences) for MS imaging by epifluorescence microscopy and image-based flow cytometry (IBFC). The mean boar MS length was 7.26 ± 0.2 µm, ranging from 6.94 ± 0.18 µm to 7.65 ± 0.31 µm. The absolute longest MS measured was 9.19 µm and the shortest was 5.83 µm. Boars in the high tertile of MS length had significantly higher conception rate (CR; *p* = 0.05) and sperm parameters. Boars within the high tertile of average number piglets born per litter had significantly shorter MS and more varied MS length than boars in the low tertile (*p* = 0.04). MS length data correlated with conventional sperm parameters including percent viable and intact acrosomes (*p* = 0.03), basal:induced oxidation ratio (measure of intracellular ROS levels; *p* = 0.02) and Comp DNA (chromatin integrity; *p* = 0.06) along with many flow cytometric AGG parameters in IBFC. Sperm head AGG intensity median absolute deviation had a negative correlation with total born (r = −0.423 *p* = 0.004). These data reveal a complex relationship between sperm MS length and aggresome abundance to sperm parameters and boar reproductive success in AI service.

## 1. Introduction

Mammalian spermatozoa carry several dozens of mitochondria organized into a mitochondrial sheath (MS), a helical structure wrapped around the sperm tail midpiece. Mitochondria contained within the MS generate ATP for dynein motor-driven movement of the sperm flagellum, which is vital for spermatozoa transport to the oocyte, but also produce reactive oxygen species (ROS) and perform other cellular functions. Length of sperm tail midpiece, occupied by the MS has been shown to correlate with reproductive traits in deer mice [1], as well as in domestic mice [2], salmon fish [3] and passerine birds [4]. However, it is not known whether this could be the case in domestic animals positioned higher on phylogenetic tree such as boars and bulls, and if so, whether such a parameter could be used as a fertility marker. The advantage of using a domestic species over wildlife for correlative studies of MS size is in the availability of extensive fertility records from hundreds, sometimes thousands of artificial insemination (AI) services. Fertility in AI is of paramount importance to agriculture and food production, and potentially relevant to human reproductive health and assisted reproductive therapy.

Studies showing a strong correlation between mitochondrial sheath length and fertility success in polygamous species indicate the importance of midpiece length in successful sperm competition. This advantage may be a result of higher energy reserves and faster swimming speeds allowing the competing sperm to reach the ova faster [5]. These reproductively competitive species include the previously mentioned domestic mice, salmon fish, and passerine birds. In primates, when compared to monandrous species, males of polyandrous species (*Erithrocebus patas* and *Pongo pygmaeus*) have been shown to have sperm with longer midpieces as well [5]. While studies suggest that evolutionary adaptation would select for longer midpiece lengths in wild polygamous species, by perusing the same parameters for domestic boars, we can understand the importance and selection for this trait for livestock fertility.

The increasing use of genomic selection favoring production traits in commercial production over reproductive performance, as well as commercialization of sexed semen in livestock artificial insemination furthers the need for more accurate andrological evaluation of AI sires. From a genetic perspective, Lush described the differences in sperm head length between X vs Y-bearing spermatozoa [6]. Since then, limited success has been achieved in correlating sperm traits to other genetic-regulated phenotypes, such as markers associated with terminally desired production traits important in all food animals. In the present study, we hypothesized that the length of sperm tail mitochondrial sheath correlates with conventional semen and sperm parameters, and with fertility in boars used for AI service. Therefore, we set out to describe the relationship between the length of the sperm tail mitochondrial sheath and fertility traits. Further, we hypothesized that other sperm phenotypes correspond with the terminal index (the genetic value for terminal production related traits).

## 2. Materials and Methods

### 2.1. General Approach

To address these questions, we developed a new technique for measurement of MS parameters based on the detection of aggresomes, the ubiquitinated protein aggregates that accumulate in the MS of mammalian spermatozoa. Sperm samples from 57 boars used for artificial insemination (AI), were incubated with ProteoStat Aggresome probe (AGG; Enzo Life Sciences, Farmingdale, NY, USA) for epifluorescence microscope imaging-morphometry and image-based flow cytometry (IBFC). ANOVA was used to assess differences between boars and Pearson’s correlation coefficients were calculated for MS length vs laboratory semen parameters.

### 2.2. Animals and Fertility Data

To assess boar fertility, Duroc boar semen was inseminated utilizing a timed-AI approach. Boars were 9-12 months of age. F1 Landrace × Yorkshire sows were administrated OvuGel^®^ (United Animal Health, Sheridan, IN, USA) 96 h post-weaning and inseminated 22 h later with 1.25 × 10^9^ sperm cells in a 35 mL dose. It is of note that conception rate of sows administrated OvuGel^®^ is calculated by total number of sows weaned, not the number of sows displaying estrus. Animals were cared for under standard operating procedures of the industry partner, The Maschhoffs LLC (Carlyle, IL, USA) in a controlled setting relevant to commercial pork production. None of the animal procedures were carried out at the University of Missouri. Relevant animal data were collected as detailed in Table 1 below. To test the % normal morphology, we counted a minimum of 100 sperm cells by phase contrast microscopy at ×1000 (100 × oil objective and 10 × eyepiece) from wet mounts of formalin-preserved samples and recorded the number of sperm with normal morphology. For the following flow cytometric assays, a Guava EasyCyte Plus with CytoSoft software was used, equipped with a 488 nm laser (Guava Technologies Inc., Millipore, Hayward, CA, USA) and a minimum of 5000 cells were analyzed. For % Mero-negative, Sybr14 staining was used as a positive sperm cell marker and the percent of spermatozoa not stained with Merocyanine 540 (M540) was determined by flow cytometry [7]. The % viable with intact acrosomes was measured by flow cytometry, analyzing a minimum of 5000 sperm cells for the percent of spermatozoa negatively stained with propidium iodide (PI) and fluorescein-labelled *Arachis hypogaea* (peanut) lectin (PNA-FITC) [8,9]. Differential staining with JC-1 was used to determine the % depolarized mitochondria, utilizing the ability of JC-1 to discriminate between mitochondria exhibiting high membrane potential from those with low membrane potential [10]. The intracellular oxidation status of viable sperm (PI-negative) was assessed by measuring the basal ROS level divided by the induced condition following incubation with hydrogen peroxide in the presence of 2’,7’-dichlorodihydrofluorescein diacetate (H2DCFDA) [11]. The Comp DNA test measures the DNA fragmentation of cells outside the main population after a timed acid stress [12,13]. To test varying fertility, we tested samples with varying semen parameters. The range of the metrics examined include: % normal morphology, 36–89%; % Mero negative, 72.6–91.8%; % depolarized mitochondria, 9.6–31.5%; % viable with intact acrosomes, 40.4–91.2; oxidation (basal:induced ratio), 0.12–1.006; and Comp DNA, 0.9–85.9%.

### 2.3. Semen Collection and Processing

Semen was collected manually using the double glove technique [14], following standard industry practices. Semen was immediately diluted in MOFA Androstar Plus^®^ semen extender within 2 °C of the ejaculate temperature and to a final concentration of 3.125 mil spermatozoa/mL. An aliquot was then shipped by overnight parcel to Kuster Research & Consulting Inc. (Atkinson, IL, USA) to analyze the semen for the above traits. A further aliquot was washed and fixed in 4% paraformaldhyde (Electron Microscopy Sciences, Hatfield, PA, USA) for 40 min, later stored in PBS (with NaN_3_) at +4 °C, and shipped by overnight parcel to University of Missouri for further analysis.

### 2.4. Mitochondrial Sheath Labelling and Measurement

Fixed sperm were sedimented on pol-L-lysine coverslips, permeabilized for 40 min in PBS + 0.1% TrX, then incubated for 10 min with 1:1000 AGG probe. Following probe incubation, coverslips were washed in PBS-TrX and images acquired in 10 random fields per boar using a 100 × oil lens on a Nikon Eclipse 800 microscope (Nikon Instruments Inc., Melville, NY, USA) with Cool Snap camera (Roper Scientific, Tuscon, AZ, USA) and MetaMorph software (Universal Imaging Crop., Downington, PA, USA). AGG fluorescence was acquired in the green channel at 500 millisecond acquisition time. The mitochondrial sheath lengths of 50 intact, grossly morphologically normal spermatozoa, were measured and recorded using the tape measure tool in MetaMorph (Version 7, Universal Imaging Corp., Downington, PA, USA).

### 2.5. Image-based Flow Cytometry (IBFC) Sample Acquisiation

Approximately 1 million spermatozoa/boar were prepared for IBFC analysis. Lower probe concentrations were necessary for IBFC than for epifluorescence microscope imaging due to camera detection differences, thus 1:2000 AGG probe and 1:1000 H33342 were used (H33342 used from the ProteoStat kit, therefore final molar concentration unknown). IBFC data acquisition was performed following previous methodology [15]. Specifically, using a FlowSight flow cytometer (FS) fitted with a 20 × microscope objective (numerical aperture of 0.9) with an imaging rate up to 2000 events per sec. The sheath fluid was PBS (without Ca^2+^ or Mg^2+^). The flow-core diameter and speed was 10 μm and 66 mm per sec., respectively. Raw image data were acquired using INSPIRE^®^ software. To produce the highest resolution, the camera setting was at 1.0 μm per pixel of the charged-coupled device. In INSPIRE^®^ FS data acquisition software, two brightfield channels were collected (channels 1 & 9), one AGG image (channel 2), one side scatter (SSC; channel 6), and one H33342 (channel 7), with a minimum of 10,000 spermatozoa collected. The following lasers and power settings were used: 405 nm (to excite H33342): 40 mW; 488 nm (to excite AGG): 50 mW; and 785 nM SSC laser: 10 mW.

### 2.6. IBFC Data Analysis

**Figure 1:**
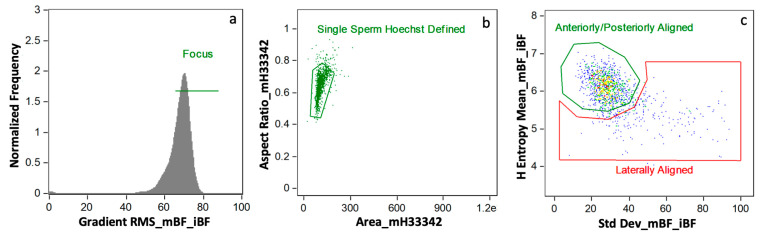
Initial Data Gating Approach. This strategy gated for cells in (**a**) focus, (**b**) single sperm cells as defined by Hoechst 33342, and (**c**) anteriorly/posteriorly aligned as opposed to laterally aligned (plot colored by density [least dense to most dense: blue, green, yellow, orange, red]).

Data were analyzed using AMNIS IDEAS^®^ analysis software version 6.2 from Luminex Corp. (Seattle, WA, USA). An initial gating approach removed distortion from final data for analysis by eliminating flow cytometric events other than single, in-focus spermatozoa (Figure 1). Briefly, this process discarded events with multiple spermatozoa, cells out of focus, and spermatozoa laterally aligned to the camera (as opposed to anteriorly/posteriorly aligned). A mask created from Hoechst 33342 (channel 7, MH33342) allowed for analyzing the sperm heads as described [9]). This combined gating and masking strategy provided robust, phenotypically clean data, ready for biomarker-based image analysis.

#### 2.6.1. Masks

**Figure 2:**
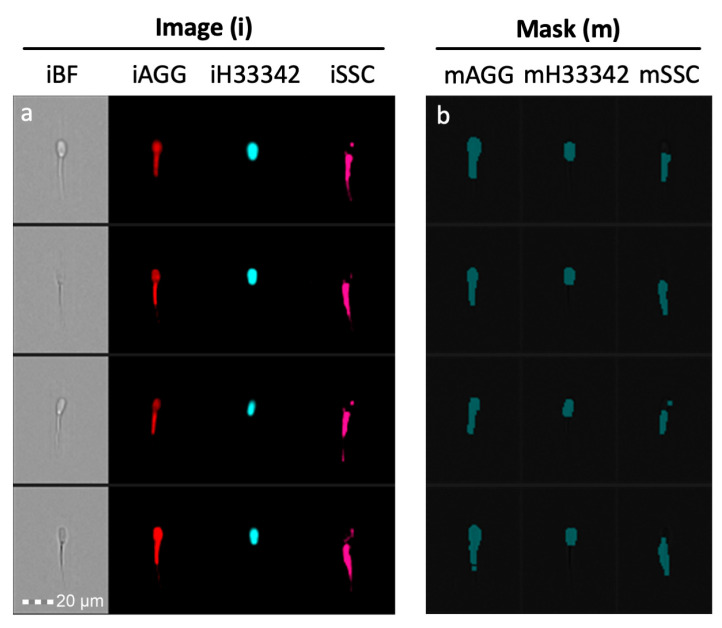
Masking Approach. (**a**) Brightfield (BF) aggresome (AGG) Hoechst 33342 (H33342) and side scatter (SSC) images of each spermatozoa were collected. (**b**) Masks that capture the fluorescent area of probe. Images are designated with an “i” and masks designated with an “m”.

Masks define the area of the cell to be analyzed. They can be created using functions (choosing an input mask and adjusting the channel and scalar input) or by combining masks using Boolean logic (IDEAS Software^®^, Version 6.3, Luminex Corp., Seattle, WA, USA). For instance, head only masks can be defined by taking the mask created by nuclear/DNA stain H33342, adding a 2-pixel dilation (since the nucleus alone is not the entire sperm head), and then analyze the image values of other fluorescent/brightfield images. Likewise, a mask for tail only can be created by subtracting this sperm head mask from the remainder of the sperm mask. Example of these masks are in Figure 2.

#### 2.6.2. IBFC Definitions

IDEAS^®^ analysis software offers over 80 base image feature calculations. When combined with multiple masks and channels, the number of image features per sperm that can be calculated are enormous. The definition of the image-based features found significant in the study are in Table S1. These features are discussed throughout as an equation: [image feature]_[mask]_[image] analyzed (e.g., Gradient RMS_mBF_iBF).

#### 2.6.3. Gating

**Figure 3:**
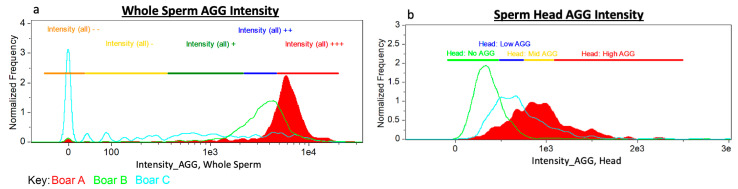
Gating Approach. Three boars with varying aggresome probe localization as examples of the gating strategy used for: (**a**) whole sperm AGG intensity and (**b**) sperm head (only) AGG intensity.

Gating was decided by observed subpopulation segregation within a single boar and between boar-to-boar differences. Examples of whole sperm and sperm head only aggresome intensity are located in Figure 3.

### 2.7. Statistics

Descriptive statistics were applied to each data set. ANOVA was used to assess differences between boars using a 2 tailed-test. Pearson’s correlation coefficients were calculated for MS length vs. conventional semen parameters, reproductive performance, and IBFC values with significance determined by a two-tailed test. Principal component analysis was performed in Weka^®^ 3.8.4 (University of Waikato, Waikato, New Zealand) and linear regression in R version 3.6.3. Weighted and unweighted (for number of matings) linear regression covariate analysis was performed using the general linear model procedure in SAS 9.4.

## 3. Results

**Figure 4:**
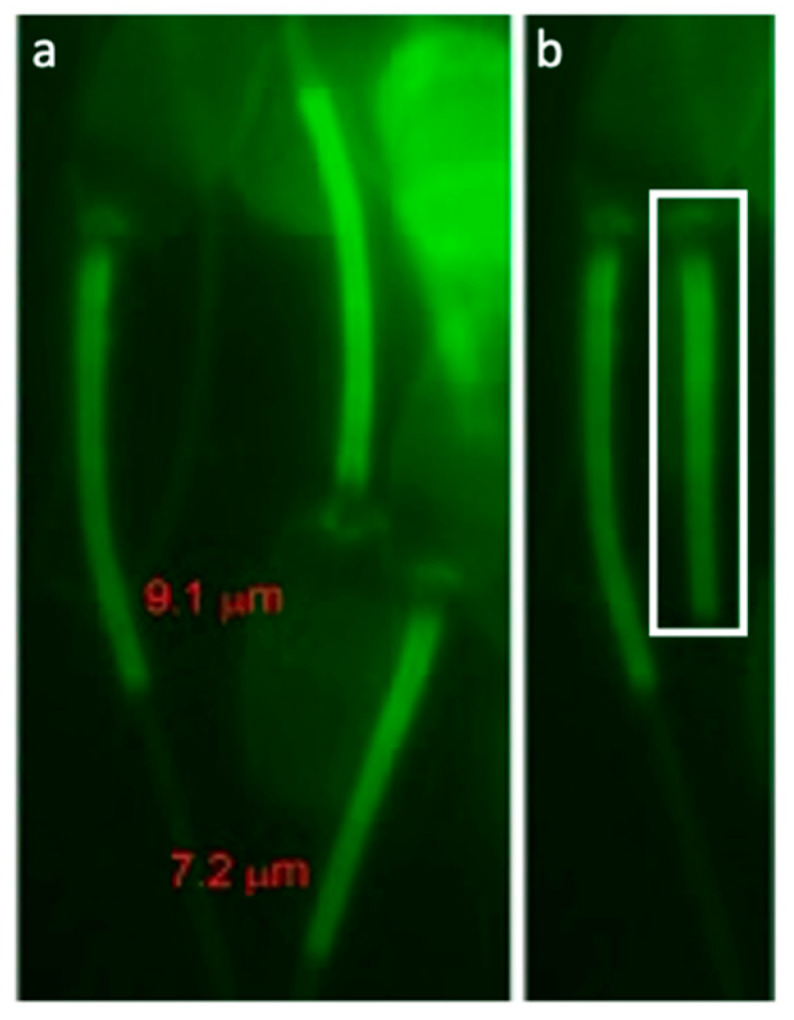
(**a**) Example image of mitochondrial sheath (MS) measurement. (**b**) Comparison of a longer MS lined up with shorter MS.

The MS of spermatozoa from 57 boars used for single sire AI were stained with ProteoStat aggresome probe for epifluorescence microscope imaging/morphometry and image-based flow cytometry (IBFC). The mean boar MS length was 7.26 ± 0.2 µm, ranging from 6.94 ± 0.18 µm to 7.65 ± 0.31 µm. The widest minimum-maximum MS length range in an individual boar was 2.45 µm, smallest range was 0.54 µm. The absolute longest MS measured in an individual spermatozoon was 9.19 µm; shortest MS on record was 5.83 µm. Example of varying MS length within a single boar is demonstrated in Figure 4. Boars in the high tertile of MS length had significantly higher conception rate (CR; *p* = 0.05) than those in the middle tertile, and also showed significantly better sperm parameters (Table 2). Boars within the high tertile of average number piglets born per litter had significantly shorter MS than boars in the low tertile (*p* = 0.04) while there was no difference in MS length when boars were divided into tertiles based on their conception rate. Boars with longer MS had more variable MS length (higher min-max range of MS lengths). There was no significant difference in covariate analysis when number of matings was weighted compared to unweighted in linear regression models for conception rate and average total born (Figures S1 and S2). Conception rate and Reproduction Index were not significantly higher in the top 10 boars with longest MS vs. 10 boars with shortest MS (*p* > 0.05).

When separated into tertiles by MS length, significant differences with conventional sperm parameters including viable and intact acrosomes (*p* = 0.03), oxidation (basal:induced ratio) (*p* = 0.02) and Comp DNA (*p* = 0.06) were discovered (Table 2). Image-based flow cytomery values were also found significantly different between various reproduction and terminal production traits (Table 3). Beside MS, the AGG intensities were contributed by aggresome labeling in the heads of morphologically abnormal spermatozoa. Additional significant correlations were found with field AI fertility, including terminal index presented throughout Table 3 and Tables S2–S5.

Building on our tertile analysis approach as done with the MS length summarized in Table 1, we expanded our analysis to include other image-based calculations of 45 boars with suitable IBFC sperm samples, sorted by various tertile groupings (group definition; Table 3). Not all 57 boars were used due to limited spermatozoa available. We found that the circularity of the sperm head/nucleus was significantly correlated (*p* = 0.005) with the 1st and 2nd tertiles of the Reproduction Index (Table 3).

**Figure 5:**
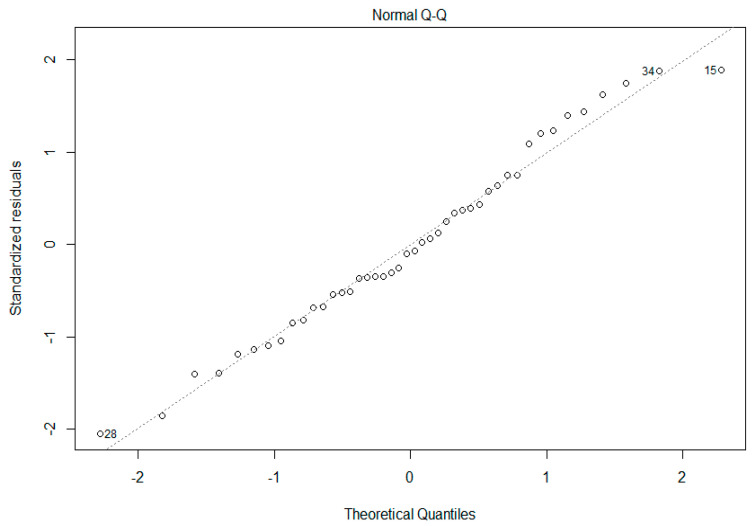
Normal Quantile-Quantile Plot. Plot showing the standardized residuals (y-axis) as a function of quantiles expected with the same mean and variance as the empirical distribution (x-axis). Individual boars (28, 34, & 15) more than 5% away from the predicted value are numerated.

A principal component analysis (PCA) was performed for dimension reduction of predicting boar terminal index with sperm traits observed by IBFC. Variables found by PCA were then used in multiple linear regression, with significant variables used in the final model. A significant regression equation was found [F(2,41)=3.415, P=0.04248] with an R^2^ of 0.1428. Boar predicted terminal index value is equal to −11.757 + 95.073 (H Entropy Std_M06_6-SSC_5, Std. Dev) + 7.126 (Diameter_M06, Mean), where (H Entropy Std_M06_6-SSC_5, Std. Dev) and (Diameter_M06, Mean) is measured in standard IBFC values. Boar terminal index value increased 95.073 points for each (H Entropy Std_M06_6-SSC_5, Std. Dev) unit of measure and 7.726 points for each (Diameter_M06, Mean) units of measure. The normal Quantile-Quantile plot is shown in Figure 5. This means current performance and genetic testing programs used to estimate potential progeny value could be augmented by including sperm traits as a more wholistic approach, with the added benefit of protecting against unintended selection for reduced reproductive fitness.

## 4. Discussion

A belief corresponding with the divergence of external fertilization to internal fertilization has been held for some time, including boar [16,17]. Specifically, a hypothesis put forward was that a selective advantage is conferred on males capable of depositing spermatozoa closer to the ova than competing males [18]. This created a sexual rather than environmental selection pressure, whereby spermatozoa that could fertilize the ova the soonest post ovulation sired the next generation. Following this, two contrasting theories for further successful sperm competition diverged: (1) an increase in sperm numbers; (2) an increase in sperm tail length (and hence energy production and velocity; reviewed in [18]). The former of the two theories predicted that spermatozoa would decrease in size while their numbers increased. These contrasting yet equally plausible theories could explain the anatomical differences among species previously found in the wild with high sperm competition: the rat (*Rattus norvegicus*) versus the boar (*Sus scrofa*). Comparatively, rat sperm flagella are longer (average 177 μm) than domesticated boar (average 36.4 μm) [19], while the number of spermatozoa per body weight are inverse (more spermatozoa in boar than rat). Recent findings confirm these two concepts are equally valuable for understanding successful sperm competition dependent on taxa: longer sperm/decreased sperm number in small endothermic animals with high metabolic constraints contrasts shorter sperm/increased sperm number in larger species where lower metabolic restraints exist [20]. Limited fertility records that can be gathered on males in wild species impose a major limitation on these considerations.

Examining relevant sperm traits in domestic boars with extensive fertility records, we found a negative correlation between mean sperm MS length and total number of piglets born per litter (SD Table 3, r = −0.299, *p* = 0.046), meaning that as the average MS length increased, fewer offspring were born per litter. This could be due to the post-fertilization processing of sperm MS with paternal mitochondrial DNA destined for degradation, to prevent deleterious effects of heteroplasmy on the embryo/fetus. We previously reported that the sperm mitochondrial sheath is degraded at the 2–4 cell stage of embryo development in bovine, murine and rhesus monkey embryos [21], but much earlier, at one cell stage in the domestic pig [22]. This process we now call post-fertilization sperm mitophagy is coregulated by autophagy and the ubiquitin-proteasome system, reviewed in [23]. Indeed, binding of ubiquitin and pro-autophagic ubiquitin-receptors to sperm MS after fertilization was documented in our previous work [24,25,26]. While longer MS length spermatozoa could produce more energy for sperm motility and theoretically higher rates of fertilization, the oocyte could have difficulties eliminating the paternal mitochondria in post-fertilization mitophagy, failing to proceed in embryogenesis. Alternatively, the decreased litter sizes with longer MS lengths could be a result of decreased percentage of spermatozoa with normal morphology as in the males with longer maximum MS lengths (SD Table 3, % Normal Morphology: Maximum MS Length, r= −0.368, *p* = 0.013).

Highlighting the value of high precision new generation flow cytometry, the IBFC side scatter and aggresome parameters were significantly correlated with traditional andrology lab mitochondrial analysis (SD Table 3). This could reflect poorer sperm survival during A.I. dose storage prior to insemination and/or poorer survival in the sow reproductive tract during the insemination to ovulation interval, warranting follow up sperm survival studies. We also found correlations with the percentage of spermatozoa with no or low to medium relative aggresome fluorescence in the sperm head was significantly associated with the basal:induced oxidation ratio (SD Table 3, r = 0.321 [no fluorescence], −0.334 [low fluorescence], −0.315 [medium fluorescence] *p* < 0.035, respectively).

Pooling semen from multiple sires is a common practice in the swine industry. A heterospermic controlled study found that up to 72% piglets in a litter can be sired by one male when two billion spermatozoa from two boars each were used to inseminate [19]. While the study found differences in the concentration of three seminal plasma proteins, motility, and acrosomal integrity, mitochondrial characteristics or MS length were not analyzed. While the current study utilized single sire AI, follow up studies could consider analyzing offspring ratios of short versus long MS sheath sires in a similar controlled heterospermic study design to elucidate theories presented here.

Other than X vs Y chromosome bearing spermatozoa, to the best of our knowledge, there have not been studies comparing other sperm phenotypes to genetic traits. Here, we show for the first-time the correlations between genetic-derived terminal index and sperm phenotypes (Table 2 and Table 3 and Figure 4). Semen traits such as sperm morphology, count, and ejaculate volume are known to vary between breeds, and it is commonly accepted throughout the swine industry that ejaculate characteristics within terminal boars often favor cross-bred boars or hybrid lines over purebreds (e.g., Duroc, Hampshire, Pietrain, Berkshire, etc.) [20]. Whether the correlations discovered here are related to poor sperm quality versus the paternal genome is not understood, but inspires future follow up studies exploring correlations between sperm phenotype to terminal index/genomic value.

## 5. Conclusions

In conclusion, the aggresome-based boar sperm analysis reveals intriguing, previously unknown relationship between exactly defined, objectively measurable sperm phenotypes and male fertility. It is a simple, rapid technique to measure MS length that could be used to study the evolution of sperm competition in wildlife, and assess current fertility, and possibly help predict future fertility in individual males and between genetic lines in livestock. Specific to boar fertility, MS morphometry it could be used to predict the need for ejaculate pooling in AI service. Additionally, MS length could inform the number sperm per dose necessary. For instance, boars with higher average MS length (decreased reproductive performance) but valuable terminal index value, could be candidates for increased sperm/dose to help compensate fertility. Future follow up studies could analyze an increased number of boars to see if these relationships are maintained in a larger sample size of various genotypes.

## Figures and Tables

**Table 1:** Animal and semen data collected for phenotype correlations.

**Animal Data**	
**Term**	**Definition**
Mean Number of Matings/Sire	The average number of sows a boar was used to breed
Terminal Index (TI)	The genetic value assigned to a boar based on specific traits (internal Maschhoffs composite value compromised of wean-to-market traits, e.g., % lean, backfat depth, etc.)
Mean Conception Rate (CR)	The number of sows confirmed pregnant at 28 day pregnancy check that were bred
Average Total Number Born/Litter (TNB)	The number of pigs born (includes live, stillborn and mummified) per litter farrowed
Reproduction Index (RI)	CR × TNB × 100
**Semen Data**	
**Term**	**Definition**
% Normal Morphology	Percent of spermatozoa with normal morphology
% Mero-negative	Percent of spermatozoa not stained with mero cyanine 540. Positive cells indicate changes in phospholipid arrangement and membrane fluidity, an early indicator of spermatozoa stress
% Viable & Intact Acrosomes	Percent viable spermatozoa with intact acrosomes not stained with propidium iodide or lectin PNA-FITC
% Depolarized mitochondria	Percent of spermatozoa with depolarized mitochondria (low membrane potential) as determined by JC-1 staining
Oxidation	Measures the intracellular level of reactive oxygen species (ROS) or free radicals in spermatozoa, calculated as the Basal:Induced ratio
Comp DNA	Measures the ability of the sperm chromatin to maintain structural integrity after undergoing acid stress

**Table 2:** Mitochondrial sheath (MS) length, fertility/AI service outcomes and laboratory semen parameters in spermatozoa of 57 boars analyzed together and within tertiles of MS length.

Parameter	All (*n* = 57)	1st TERTILE (Low/Shortest)	2nd TERTILE (Middle)	3rd TERTILE (High/Longest)
Mean MS length ± SD (µm)	7.26 ± 0.2	7.12 ± 0.09	7.26 ± 0.03	7.4 ± 0.1
Mean MS length range (µm)	1.01 ± 0.4	1.04 ± 0.4	0.85 ± 0.29	1.12 ± 0.46
Mean shortest MS (µm)	6.73 ± 0.4	6.55 ± 0.29	6.79 ± 0.29	6.86 ± 0.25
Mean longest MS (µm)	7.74 ± 0.33	7.59 ± 0.26	7.64 ± 0.09	7.97 ± 0.43
Mean number of matings/sire	113.27 ± 105.46	104.79 ± 93.15	91.39 ± 59.21	142.21 ± 141.21
Terminal index	129.23 ± 6.83	129.64 ± 7.11	128.47 ± 5.88	129.53 ± 7.65
Mean conception rate (% CR)	74.55 ± 1.0	74.79 ± 9.96	71.0 ± 13.3	77.73 ± 6.02 ^p23^ *
Average total number born/liter	13.72 ± 0.74	13.92 ± 0.71	13.61 ± 0.79	13.62 ± 0.7
Reproduction index	1024.18 ± 161.9	1041 ± 149.56	969.61 ± 205.2	1059.47 ± 109.11
% Normal morphology	71.39 ± 13.22	72.18 ± 12.11	71.03 ± 12.65	71.44 ± 15.58
% Mero-negative	85.89 ± 4.36	87.18 ± 2.85	84.62 ± 4.86	85.6 ± 4.92
% Depolarized mitochondria	18.12 ± 5.61	18.24 ± 2.85	17.61 ± 6.02	18.01 ± 4.41
% Viable & intact acrosomes	72.94 ± 13.92	75.27 ± 10.86	66.34 ± 20.17	76.72 ± 5.41 ^p23^ *
Oxidation (Basal:Induced ratio)	0.44 ± 0.25	0.54 ± 0.3	0.35 ± 0.22 ^p12^ *	0.44 ± 0.17
Comp DNA	5.37 ± 11.97	2.29 ± 0.94 ^p12 x^	11.2 ± 19.55	2.46 ± 0.96 ^p23 x^
Number of matings per tertile	6535	1991	1842	2702

Significance: * = 0.05; ^x^ = 0.1. ^p12^ = *p*-value between 1st and 2nd tertile; ^p23^ = *p*-value between 2nd and 3rd tertile.

**Table 3:** Assorted IBFC/MS length sperm values, fertility/AI service outcomes and laboratory semen parameters with significance found in spermatozoa of 45 boars analyzed within specified groupings.

PARAMETER	ALL (*n* = 45)	1st TERTILE (Low)	2nd TERTILE (Middle)	3rd TERTILE (High)	Grouping
Sperm head/nucleus circularity, Mean (FlowSight units)	4.91 ± 0.38	5.11 ±0.47	4.71 ± 0.23 ^p12^ **	4.90 ± 0.311	Tertile: Reproduction Index ^1^
Average total number born/litter	13.77 ±0.74	14.31 ± 0.37 ^p12^ ***	13.5 ± 0.73	13.85 ± 0.79	Tertile: MS length ^2^
Average total number born/litter	13.77 ± 0.74	14.05 ± 0.51	13.82 ± 0.70 ^p23^ **	12.85 ± 0.1 ^p13^ **	Tertile: Head AGG Bright Detail Intensity R3 ^3^
% Viable with intact acrosome	76.50 ± 8.45	78.88 ± 4.70	77.28 ± 7.17 ^p23^ **	66.42 ± 15.15 ^p13^ **	Tertile: Head AGG Bright Detail Intensity R3 ^4^
% Intensity AGG +++	8.37 ± 7.68	15.99 ± 13.06 ^p13^ **	5.94 ± 2.99 ^p23^ **	6.5 ± 3.18	Tertile: Terminal Index ^5^
Oxidation (Basal:Induced ratio)	0.47 ± 0.25	0.30 ± 0.11	0.50 ± 0.22 ^p12^ **	0.65 ± 0.31 ^p13^ **	Tertile: Terminal Index ^5^
Oxidation (Basal:Induced ratio)	0.47 ± 0.25	0.53 ± 0.28 ^p12^ *	N/A	0.39 ± 0.18	Quantile: MS length ^6^
Side Scatter Symmetry_3, Mean	12.15 ± 0.50	12.45 ± 0.45 ^p13^ **	12.06 ± 0.42 ^p12^ **	11.89 ± 0.55	Tertile: Terminal Index ^5^
H Correlation Mean Side Scatter_5, Std. Dev.	0.199 ± 0.0096	0.205 ± 0.0123 ^p13^ *	0.197 ± 0.0064 ^p23^ *	0.193 ± 0.0064	Tertile: Terminal Index ^5^

Significance: *** = 0.001; ** = 0.01; * = 0.05. ^p12^ = *p*-value between 1st & 2nd tertile; ^p23^ = *p*-value between 2nd & 3rd tertile; ^p13^ = *p*-value between 1st & 3rd tertile; Grouping: ^1^ = Evenly split: High/medium/low; ^2^ = Separation: 7.20, 7.40; ^3^ = Median absolute deviation (MAD), Gating: Head w/High AGG, Separation: 200, 400; ^4^ = MAD, Head: High AGG, Separation: 200, 400; ^5^ = Separation: 125 & 135; ^6^ = Separation: 7.30.

## Supplementary Materials

The following are available online at https://www.mdpi.com/2076-3921/9/11/1033/s1, Table S1: Image Feature Definitions, from IDEAS^®^ Software Version 6.3 (Luminex Corp., Seattle, WA, USA), Table S2: Correlations of semen parameters found not significant, Table S3: Assorted correlations of IBFC with fertility/AI service outcomes and laboratory semen parameters found in spermatozoa of 45 boars, Table S4: Correlations of terminal index with fertility/AI service outcomes and laboratory semen parameters not found significant corresponding with manuscript Figure 5, Table S5: Correlations of terminal index with fertility/AI service outcomes and laboratory semen parameters with significance found in spermatozoa of 45 boars, Figure S1: The following graphs shows the scatter plot of conception rate by each covariate together with regression lines unweighted (blue) and weighted (orange) by logarithmic function of number of matings. Each individual circle represents a boar and is colored by blue gradient corresponding to number of matings. There are no significant differences between unweighted and weighted regression lines, Figure S2: The following graphs shows the scatter plot of average total born by each covariate together with regression lines unweighted (blue) and weighted (orange) by logarithmic function of number of matings. Each individual circle represents a boar and is colored by blue gradient of number of matings. There are no significant differences between unweighted and weighted regression lines.
