# Supplementary material for: Relationship between the Length of Sperm Tail Mitochondrial Sheath and Fertility Traits in Boars Used for Artificial Insemination
Source: Antioxidants (Basel). 2020 Oct 23;9(11):1033. doi: 10.3390/antiox9111033 (PMC7690677; doi:10.3390/antiox9111033)

Supplemental Data File

Table S1. Image Feature Definitions, from IDEAS ® Software Version 6.2 (Luminex Corp., Seattle, WA, USA).

Image Feature	Definition
Angle Intensity	Angle is the angle of the major axis from a horizontal plane in radians.
Area	The number of microns squared in a mask is equal to the Area. 1 pixel = 1.0 μm².
Aspect Ratio	Aspect Ratio is the Minor Axis divided by the Major Axis and describes how round or oblong an object is.
Aspect Ratio Intensity	Aspect Ratio Intensity is the Minor Axis Intensity divided by the Major Axis Intensity.
Bright Detail Intensity R3	The Bright Detail Intensity R3 feature computes the intensity of localized bright spots within the masked area in the image. The intensity of bright spots that are 3 pixels in radius or less are included and the local background around the spots is removed before the intensity computation.
Centriod Y	Centroid Y is the number of pixels in the vertical axis from the upper, left corner of the image to the center of the mask.
Circularity	Circularity measures the degree of the mask's deviation from a circle. Its measurement is based on the average distance of the object boundary from its center divided by the variation of this distance. Thus, the closer the object to a circle, the smaller the variation and therefore the feature value will be high. Vice versa, the more the shape deviates from a circle, the higher the variation and therefore the Circularity value will be low.
Diameter	The Diameter feature provides the diameter of the circle that has the same area as the object.
Gradient RMS	The Gradient RMS feature measures the sharpness of an image by detecting large changes of pixel values in the image. It is computed using the average gradient of a pixel normalized for variations in intensity levels.
H Correlation	The Haralick (H) texture features are a set of texture features based on the second order statistics computed from the joint 2-D probability distribution of pixel intensities in the image. This distribution is referred to as the Gray Level Co-occurrence Matrix (GLCM). Correlation measures how similar pixel pairs are, and is the opposite of contrast. Images with high correlation are very uniform and lack variant texture.
H Energy	Energy is a measure of intensity concentration in the cell. At one extreme is the case of a uniform distribution with all probabilities being equal. This image has several intensity variations with no noticeable concentration of high intensity and thus has low energy. At the other extreme is the case of a very narrow distribution with a few elements having high values. This image will have notable intensity concentrations and thus, has high energy.
H Entropy	Entropy is also a measure of high intensity concentration in the cell. However, this feature relates to the randomness of the intensities in the image. Images that have distinct areas of intensity concentration are less random and thus, have low entropy. Images that have a range of equally likely intensity pairings have less distinct intensity concentrations, and correspondingly, have higher entropy. Entropy is the opposite of energy.
Height	Using the bounding rectangle, Height is the number of microns of the longer side.
Length	Length measures the longest part of an object. Unlike the Major Axis feature, Length can measure the object's length even if it folds to form a cashew, banana, or doughnut shape, where in many of these cases the major or minor axis features would not be able to differentiate these with true circular shaped objects with no hole.
Symmetry 3	The Symmetry 3 feature measures the tendency of the object to have a three-fold axis of symmetry.

Table S2. Correlations of semen parameters found not significant.

PARAMETERS	*r*	*P-value*
Not Significant:		
% Normal Morphology: Conception Rate	*0.206*	*0.175*
% Normal Morphology: Total Born	*0.001*	*0.994*
% Mero Negative: Conception Rate	*-0.033*	*0.828*
% Mero Negative: Total Born	*0.286*	*0.057*
% Depolarized Mitochondria: Conception Rate	*-0.017*	*0.911*
% Viable with Intact Acrosome: Conception Rate	*0.219*	*0.148*
% Viable with Intact Acrosome: Total Born	*0.266*	*0.078*
Oxidation (Basal:Induced Ratio):Conception Rate	*-0.078*	*0.612*
Oxidation (Basal:Induced Ratio):Total Born	*0.064*	*0.676*
CompDNA: Conception Rate	*-0.062*	*0.685*
Mean Length MS: Conception Rate	-0.038	0.806
Std Dev Length MS: Conception Rate	0.074	0.628
Std Dev Length MS: Total Born	-0.264	**0.080**
Range Length MS: Conception Rate	0.117	0.444
Range Length MS: Total Born	-0.177	0.244
Minimum MS Length: Conception Rate	-0.227	0.133
Minimum MS Length: Total Born	-0.085	0.578
Maximum MS Length: Conception Rate	-0.113	0.461
% Depolarized Mitochondria: Minimum MS Length	0.067	0.662
% Depolarized Mitochondria: Maximum MS Length	0.146	0.339
% Viable with Intact Acrosome: Minimum MS Length	-0.241	0.111

Table S3. Assorted correlations of IBFC with fertility/AI service outcomes and laboratory semen parameters found in spermatozoa of 45 boars.

PARAMETERS	*r*	*P-value*
**Fertility Related**		
Bright Detail Intensity R3_mH33342_iAGG, Median Absolute Deviation (MAD), Head High AGG: Total Born	*-0.423*	***0.004***
**Sperm Trait Related**		
H Energy Mean_mSSC_iSSC_5, Median: % Viable with Intact Acrosome	*0.621*	***< 0.00001***
Centroid Y Intensity_mSSC_iSSC, MAD: % Viable with Intact Acrosome	*-0.607*	***< 0.00001***
Bright Detail Intensity R3_mSSC_iSSC, Median: % Viable with Intact Acrosome	*0.531*	***0.0002***
Bright Detail Intensity R3_mH33342_iAGG, Median, Head High AGG: CompDNA	*0.496*	***0.0005***

Bright Detail Intensity R3_mH33342_iAGG, Mean, Head: Low AGG: Mean Length MS	*0.442*	***0.002***
Symmetry 3_mSSC_iSSC, Median: % Normal Morphology	*0.441*	***0.002***
Length_mH33342, Mean: % Viable with Intact Acrosome	*0.420*	***0.004***
Height_mH33342, Mean: % Viable with Intact Acrosome	*0.413*	***0.005***
% Head No AGG [gated]: % Viable with Intact Acrosome	*-0.377*	***0.011***
% Head: Mid AGG [gated]: % Viable with Intact Acrosome	*0.351*	***0.018***
% Head Low AGG [gated]: % Viable with Intact Acrosome	*0.339*	***0.023***
**Mitochondria Related**		
Symmetry 3_mSSC_iSSC, Std. Dev.: % Depolarized Mitochondria	*0.650*	***0.000002***
Aspect Ratio_mSSC, MAD: % Depolarized Mitochondria	*0.516*	***0.0003***
Angle Intensity_mSSC_iSSC, Mean: % Depolarized Mitochondria	*-0.515*	***0.0003***
Bright Detail Intensity R3_mSSC_iSSC, Median: % Depolarized Mitochondria	*-0.477*	***0.001***
Symmetry 3_mSSC_iSSC, Mean: Oxidation (Basal:Induced Ratio)	*-0.440*	***0.002***
Area_mSSC, Median: % Depolarized Mitochondria	*-0.406*	***0.006***
Aspect Ratio Intensity_mSSC_iSSC, Mean: % Depolarized Mitochondria	*0.403*	***0.006***
% Head: No AGG [gated]: Oxidation (Basal:Induced Ratio)	*0.321*	***0.032***
% Head Low AGG [gated]: Oxidation (Basal:Induced Ratio)	*-0.334*	***0.025***
% Head: Mid AGG [gated]: Oxidation (Basal:Induced Ratio)	*-0.315*	***0.035***
Semen Parameter Related		
CompDNA: Total Born	*-0.327*	***0.028***
% Depolarized Mitochondria: Total Born	*-0.322*	***0.031***
Mean Length MS: Total Born	*-0.299*	***0.046***
Maximum MS Length: Total Born	*-0.322*	***0.031***
Mean Length MS: Oxidation (Basal:Induced Ratio)	*-0.464*	***0.001***
% Normal Morphology: Maximum MS Length	*-0.368*	***0.013***
Not Significant:		
Circularity_mH33342, Mean: Conception Rate	*-0.233*	*0.123*
Circularity_mH33342, Mean: Total Born	*-0.210*	*0.165*

Table S4. Correlations of terminal index with fertility/AI service outcomes and laboratory semen parameters of interest that were not found significant corresponding with manuscript **Figure 5**.

PARAMETERS	***r***	***P-value***
**Sperm Trait Related**		
Not Significant:		
CompDNA: Terminal Index	*0.202*	*0.183*
% Mero Negative: Terminal Index	*0.142*	*0.351*
% Depolarized Mitochondria: Terminal Index	*-0.223*	*0.142*
% Normal Morphology: Terminal Index	*-0.041*	*0.787*
% Viable with Intact Acrosome: Terminal Index	*-0.037*	*0.809*

**Fertility Related**		
Not Significant:		
Total Born: Terminal Index	*0.071*	*0.600*
Conception Rate: Terminal Index	*0.036*	*0.812*

Table S5. Correlations of terminal index with fertility/AI service outcomes and laboratory semen parameters with significance found in spermatozoa of 45 boars.

PARAMETERS	***r***	***P-value***
Oxidation (Basal:Induced Ratio):Terminal Index	*0.438*	***0.003***
H Correlation Mean_mSSC_iSSC_5, Std. Dev.: Terminal Index	*-0.424*	***0.004***
Symmetry 3_mSSC_iSSC, Mean: Terminal Index	*-0.422*	***0.004***

Figure S1. The following graphs shows the scatter plot of conception rate by each covariate together with regression lines unweighted (blue) and weighted (orange) by logarithmic function of number of matings. Each individual circle represents a boar and is colored by blue gradient corresponding to number of matings. There are no significant differences between unweighted and weighted regression lines.

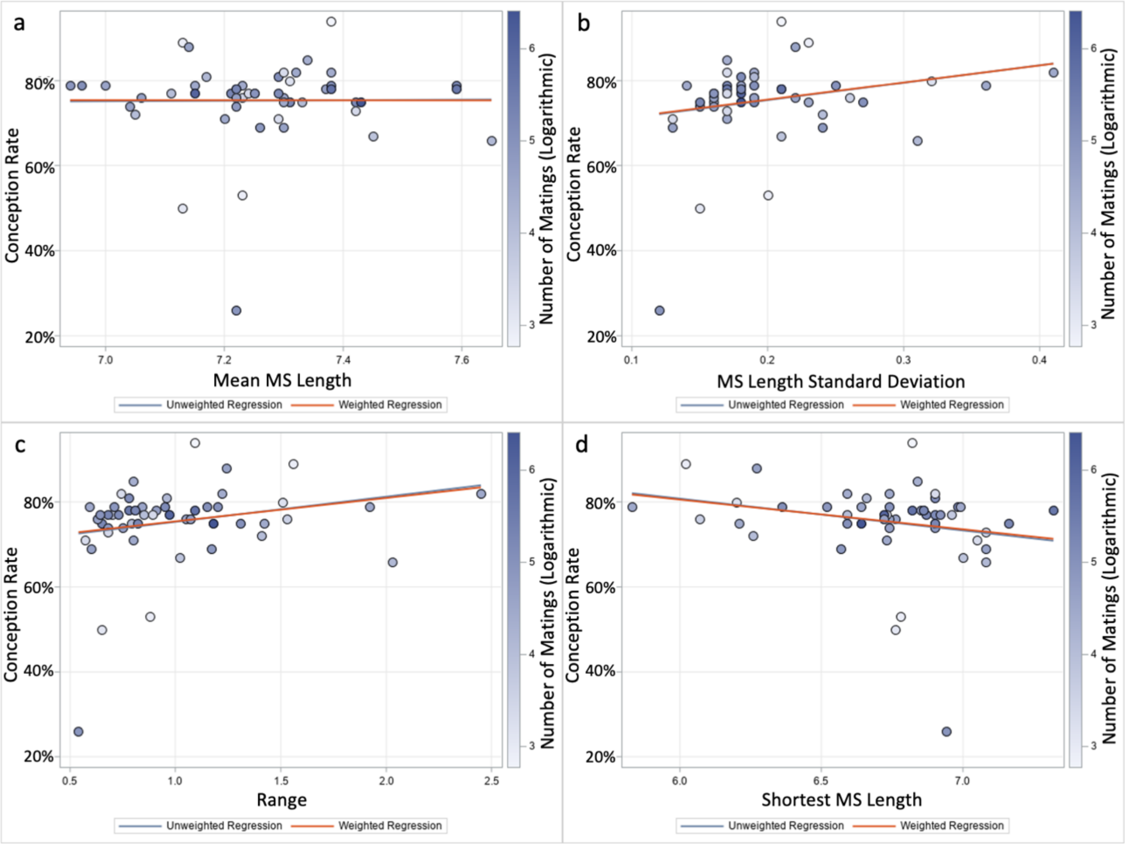

Figure S2. The following graphs shows the scatter plot of average total born by each covariate together with regression lines unweighted (blue) and weighted (orange) by logarithmic function of number of matings. Each individual circle represents a boar and is colored by blue gradient of number of matings. There are no significant differences between unweighted and weighted regression lines.

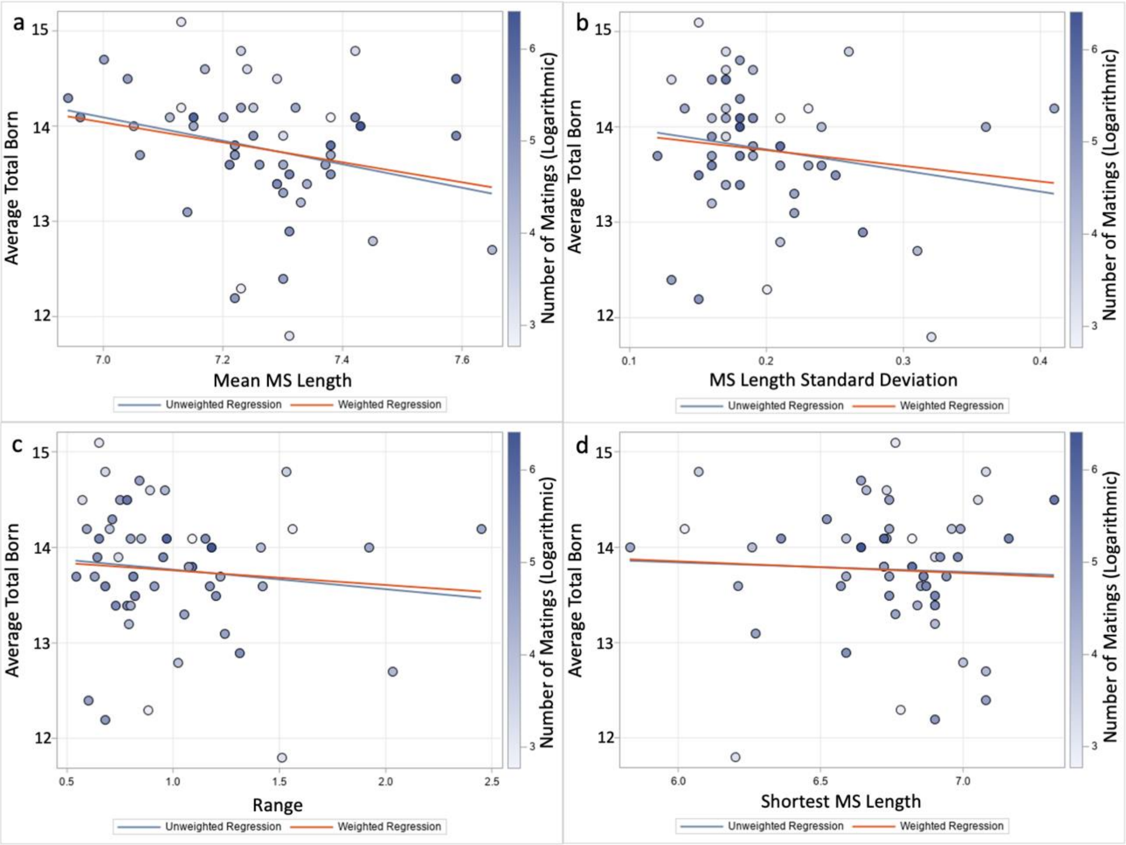

Supplement: Supplementary file 1 [file antioxidants-09-01033-s001.pdf]
